# *Gynura divaricata* Water Extract Presented the Possibility to Enhance Neuronal Regeneration

**DOI:** 10.1155/2021/8818618

**Published:** 2021-02-17

**Authors:** Fahsai Kantawong, Chanakarn Saisuwan, Pirakorn Soeratanapant, Phenphichar Wanachantararak, Jiang Nan, Jianming Wu, Young-Tae Chang

**Affiliations:** ^1^Department of Medical Technology, Faculty of Associated Medical Sciences, Chiang Mai University, Chiang Mai 50200, Thailand; ^2^The Dental Research Center, Faculty of Dentistry, Chiang Mai University, Chiang Mai 50200, Thailand; ^3^School of Pharmacy, Southwest Medical University, Luzhou 646000, China; ^4^Center for Self-assembly and Complexity, Institute for Basic Science (IBS), Pohang 37673, Republic of Korea; ^5^Department of Chemistry, Pohang University of Science and Technology (POSTECH), Pohang 37673, Republic of Korea

## Abstract

*Gynura divaricata* (GD) is an Asian herb widely used as an alternative medicine and functional food for type 2 diabetes. Diabetic neuropathy is considered as an important complication of diabetic patients. This study focused on neuroregenerative effects of GD for use in the prevention of diabetic neuropathy. GD leaves were cut and boiled in water to mimic real-life cooking. The boiled content was filtered through white gauze and lyophilized to preserve as dried powder. Antioxidant assay was performed using DPPH assays. UHPLC-QTOF-MS/MS was employed to test for important compounds in the extract of these herbs. MTT assay was used to test for cell viability. The extracts at concentration of 250 *μ*g/mL were tested with human gingival cell to observe the change of gene expression. The DPPH assay showed that GD water extract at the concentration of 5000 *μ*g/mL could inhibit DPPH radical for 39.2%. The results showed that 5000 *µ*g of GD water extract contained total phenolic content equivalent to 310.9 *µ*g standard gallic acid. UHPLC-QTOF-MS/MS result found phenolic acids and flavonoids as the main components. Human gingival cells treated with 250 *μ*g/mL of GD water extract for 10 days showed upregulation of some neuronal differentiation markers. Staining with Cdr3 dye confirmed the presentation of neuronal progenitors. The extract at the concentration of 250 *μ*g/mL was also tested with apical papilla cells to screen for change of gene expression by RNA sequencing. The result also showed significant upregulation of alpha-internexin (*INA*). These results indicated that GD water extract might have an inductive effect for neural regeneration and could be used as functional food and supplementation for the prevention or treatment of diabetic neuropathy. This work provided the basic knowledge for further investigations into the benefits of GD for diabetic neuropathy.

## 1. Introduction

Asian people used *Gynura divaricata* (Bai Bei San Qi in China) as food for a long time, but the medicinal properties that affected global gene expression in primary human cells were not reported. In Taiwan, GD was a traditional medicine widely used to treat liver diseases such as hepatitis and liver cancer. GD extracts made hepatocellular carcinoma cells more sensitive to cisplatin, and GD water extracts could inhibit the growth of cancer stem cells [1]. GD was a traditional Chinese medicine which could be used to treat bronchitis, pulmonary tuberculosis, whooping cough, sore eyes, toothache, and osteoarthritis [2].

In China, this herb was approved by the Minister of Public Health of the People's Republic of China in 2010 as a natural medicine used to treat diabetes. GD received a lot of attention as it has been used as a folk medicine to treat diabetes in Jiangsu, Zhejiang, and Sichuan, southern China. Infusion tea made from fresh leaves of GD was found to have excellent hypoglycemic effects [3]. Eating dry powder made from the leaves and stems of GD could lower blood glucose levels in mice via insulin signaling [4]. Study of phytochemical substances of this plant showed the relationship between hypoglycemic and natural compounds in this herb. Previous study reported 2 types of toxic alkaloid pyrrolizidine [5], while other studies found cerebrosides and flavonoids [6–8]. The experiments in mice indicated that GD water extract could reduce blood glucose and lipid levels and could control bodyweight of rats. In addition, the GD extract significantly increased sugar intake into HepG2 which were insulin-resistant cells. GD water extract inhibited NF-*κ*B activation and reduced insulin pathway deficiencies such as IRS1, AKT, and GLUT1 [9]. The stems of GD contained dicaffeoylquinic acid and chlorogenic acid which reduced apoptosis of pancreatic cells in diabetic patients, therefore, helping to alleviate the severity of diabetes [3, 10]. 3, 5−/4, 5-Dicaffeoylquinic acid and chlorogenic acid reduced islet cell apoptosis and improved pancreatic function in type 2 diabetic mice [3].

Peripheral nervous complications were the most common leading causes to disability which had a big economic impact on caring of diabetic patients. More than half of people with diabetes mellitus developed neuritis. It was also the leading cause of deterioration in quality of life due to pain and sensory loss [11]. Most of the GD studies focus on reducing blood sugar levels [9, 12–15]. No research has focused on the use of GD for the prevention and supplementation in diabetic neuropathy, the most common complication in diabetes mellitus [16]. Diabetic neuropathy was a neurodegenerative disorder of the peripheral nervous system that preferentially targets sensory axons caused by persistent hyperglycemia, microvascular insufficiency, and oxidative stress [17]. The researcher therefore hypothesized that GD water extract which contained high phytochemical constituents and antioxidants should be able to consume as functional food for diabetic neuropathy.

## 2. Methods

### 2.1. Preparation of Herb Extracts

**Figure 1:**
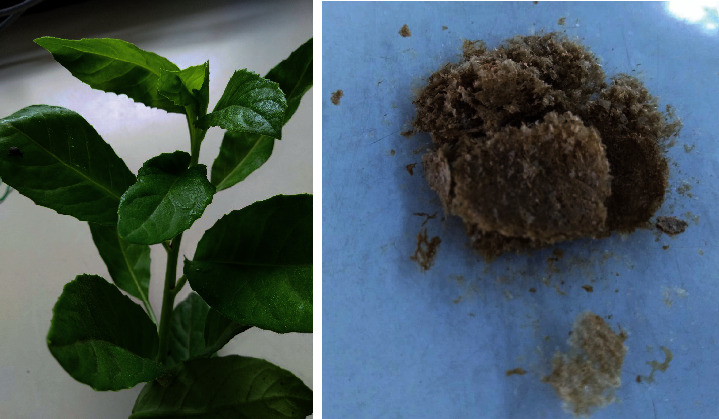
GD water extract. Fresh GD and GD water extract powder obtained after lyophilized.

**Figure 2:**
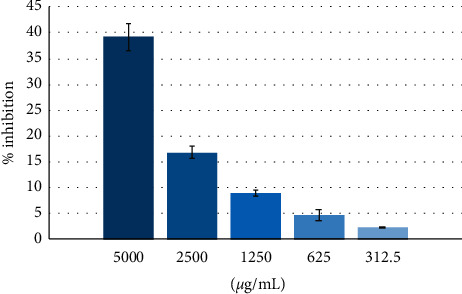
DPPH assay. Antioxidant activity of GD extract at various concentrations. Triplicate controls and tests were prepared for each concentration, and % inhibition was presented in the form of mean ± SD.

Fresh GD was purchased from local market in Chiang Mai University, Thailand (the GPS location is indicated in Figure 1S). The plant material was previously identified by J. F. Maxwell from the Department of Biology at CMU. A voucher specimen (herbarium no. N. Jiangseubchatveera 2) was deposited at the CMU herbarium of the Department of Biology [18]. Only leaves of GD were used in this study, and the stems were discarded. After purchasing, GD leaves were cleaned in tap water, and 700 grams of GD leaves were cut into small pieces and boiled in distilled water 1400 mL for 5 minutes. After that, the boiled solution was cooled down at 4°C overnight. The next day, the solution was filtered through white gauze. The filtrate was aliquoted in lyophilized flasks and frozen at −20°C before lyophilized into powder for 7 days (plant material processing is shown in Figure 2S). The lyophilized powder was stored at −20°C before use.

### 2.2. Total Phenolic Content (Folin-Ciocalteu Assay)

Dried extract was dissolved in distilled water to the concentration of 5000 *µ*g/mL. Then, 50 *µ*L of GD solution was mixed with 2.5 mL Folin-Ciocalteau reagent, and 2.0 Na_2_CO_3_ was added. The reaction was incubated at 45°C in a waterbath for 15 minutes, and the optical density was measured at 764 nm. A standard curve was prepared by diluting gallic acid to the concentrations of 10000, 5000, 2500, 1250, 625, and 312.5 *μ*g/mL, and then, the same test as described for the GD extract was performed. 2.5 mL of Folin-Ciocalteau reagent mixed with 2.0 mL Na_2_CO_3_ was used as blank. The concentration of phenolic content was calculated from the standard curve. The experiment was repeated three times, and the mean was calculated to report as total phenolic content of GD solution at a concentration of 5000 *µ*g/mL.

### 2.3. Antioxidant Capacity (DPPH Assay)

Dried extract was dissolved in distilled water to the concentrations of 5000, 2500, 1250, 625, and 312.5 *µ*g/mL. After that, 50 *µ*L of each concentration was mixed with 2950 *µ*L of DPPH reagent (90 *µ*g/mL) and incubated in the dark at room temperature for 15 minutes. A standard curve was prepared by diluting ascorbic acid to the concentrations of 10000, 5000, 2500, 1250, 625, and 312.5 *μ*g/mL and then performed the same test as described for the GD extract. The absorbances were measured at 515 nm, and the optical densities were calculated for % inhibition using the following equation. Triplicate controls and tests were prepared for each concentration, and % inhibition was presented in the form of mean ± SD.

(1) $$\begin{array}{c} \text{\% inhibition}=\frac{\text{Absorbance control}-\text{ absorbance test}}{\text{Absorbance control}}\;\times \;100. \end{array}$$

### 2.4. UHPLC-QTOF-MS/MS Analyses

The UHPLC analysis was carried out on an AB Sciex ExionLC system (AB SCIEX, Foster City, CA, USA), equipped with the ExionLC solvent delivery system, ExionLC AD Autosampler, ExionLC AD Column oven, ExionLC Degasser, ExionLC AD Pump, ExionLC PDA Detector, and ExionLC Controller. The analytical column was a Shim-pack XR-ODSII column (2.0 mmi d × 75 mm). The column oven temperature was set at 30°C. The mobile phases consisted of water containing 0.1% formic acid (solvent A) and acetonitrile (solvent B). The flow rate was set at 0.3 mL/min. The binary gradient was applied with linear interpolation as follows: 18.00 min for 45.0% B, 23.00 min for 100.0% B, 24.00 min for 100.0% B, 24.01 min for 5.0% B, and 27.00 min for 5.0% B. The UHPLC-QTOF-MS/MS detection was conducted on a Sciex QTOFTM X500R system with a TurboIonSpray® source both in the positive and negative electrospray ion modes (AB SCIEX, Foster City, CA, USA). The parameters of the electrospray ionization applied in the positive mode were ion spray voltage 5500V, ion source temperature 550°C; curtain gas 35 psi, ion source gas 1 (GS 1) 55 psi, ion source gas 2 (GS 2) 55 psi, and declustering potential (DP) 50V. The mass ranges were set at m/*z* 60–2000 Da for the TOF-MS scan and 50–2000 Da for the TOF-MS/MS experiments. In the IDA-MS/MS experiment, the collision energy (CE) was set at 35 eV, and the collision energy spread (CES) was 0 eV for the UHPLC-QTOF-MS/MS detection. The parameters of the electrospray ionization applied in the negative mode were ion spray voltage −4500V, ion source temperature 550°C, curtain gas 35 psi, ion source gas 1 (GS 1) 55 psi, ion source gas 2 (GS 2) 55 psi, and declustering potential (DP) −80V. The mass ranges were set at m/*z* 60–2000 Da for the TOF-MS scan and 50–2000 Da for the TOF-MS/MS experiments. In the IDA-MS/MS experiment, the collision energy (CE) was set at −35 eV, and the collision energy spread (CES) was 0 eV for the UHPLC-QTOF-MS/MS detection. The most intensive 10 ions from each TOF-MS scan were selected as MS/MS fragmentation. Dynamic background subtraction (DBS) was applied to match the information-dependent acquisition (IDA) tests for UHPLC-QTOF-MS/MS detection. The LC-MS/MS data were analyzed using PeakView® 1.2 software (AB SCIEX, Foster City, CA, USA).

### 2.5. Cell Viability Study (MTT Assay)

Human gingival cells were obtained according to a protocol approved by the Ethics Committee, Faculty of Dentistry, Chiang Mai University (68/2019). Human gingival cells (passage 3) were seeded into a 12-well plate at the density of 20000 cells/well in complete DMEM. GD extract was dissolved in complete DMEM at the concentrations of 0, 100, 250, and 500 *μ*g/mL. Cells were left in the CO_2_ incubator for 1 day before treatment with various concentrations of GD extract. Cells were incubated in the CO_2_ incubator for 48 hours. Human gingival cells cultured in normal complete DMEM were used as controls. When the incubation time was over, media was discarded and replaced with 2 mL of media containing MTT (1 mg/mL) before incubating for 2 hours in the CO_2_ incubator. After the media was discarded, 2 mL of DMSO was added to dissolve the formazan crystals. The absorbance was measured at 570 nm and 630 nm. The optical densities used to calculate for % cell viability was presented in the following equation. Triplicate treatments were performed for each concentration. For MTT assay, controls and treatments were usually carried out in triplicate for each concentration. Cell viability of each concentration was presented in the form of mean ± SD. Cell viability of each concentration was compared to control using the independent *t*-test in SPSS 17.0.

(2) $$\begin{array}{c} \text{\% Cell viability }=\frac{\text{Absorbance test }570-\text{ absorbance test }630}{\text{Absorbance control }570-\text{ absorbance control }630}\times 100. \end{array}$$

### 2.6. Human Gingival Cell Culture

Human gingival cells were obtained according to a protocol approved by the Ethics Committee, Faculty of Dentistry, Chiang Mai University (68/2019). Human gingival cells (*n* = 2; passages 3–5) were seeded into a 6-well plate at the density of 40000 cells/well and incubated in CO_2_ incubator for 24 hours. In the next day, media was discarded and replaced with complete DMEM containing 250 *µ*g/mL GD extract. Human gingival cells (passages 3–5) at the density of 40000 cells/well cultured in plain complete DMEM were used as control group. Cultured media was changed every 3 days. Cells were cultured for 3 days, 10 days, and 14 days. The experiment was divided into 2 groups: the control group (cells cultured in complete DMEM) and the treated group (cells cultured in DMEM containing 250 *µ*g/mL GD extract). Triplicate control samples and triplicate test samples were prepared for each timepoint.

### 2.7. Real-Time PCR

Total RNA amounts were measured with Nanodrop 2000. The reaction solutions were prepared with 6 *µ*L of RNA, 4 *µ*L of 5x RT Mastermix, and 10 *µ*L of DEPC water. The RNA was converted to cDNA in the thermocycler using the following program: 37°C for 15 minutes, 50°C for 5 minutes, and 98°C for 5 minutes. cDNA was stored at −20°C. NO-RT control was prepared with the same method using 5x RT buffer. Real-time PCR reaction was prepared with 8 *µ*L RNA, 10 *µ*L SYBR Green Mastermix, and 2 *µ*L primer (the final concentration of each the primer was 1.0 *µ*M) in the total volume of 20 *µ*L. The PCR program was set as following: (1) preincubation at 95°C for 2 minutes. (2) PCR consisted of denature at 95°C, annealing at 60°C, and extension at 72°C. Real-time PCR was performed for 40 cycles. The reference gene, GAPDH was used for normalization. The sequences of primers used in this study are presented in Table 1S. The experiment was divided into 2 groups: the control (cells cultured in complete DMEM) and the treatment (cells cultured in DMEM containing 250 *µ*g/mL GD water extract). Triplicate control samples and triplicate test samples were prepared. Fold change of gene expressions is presented in the form of mean ± SD. The independent *t*-test (SPSS 17.0) was used to determine the difference between groups. Statistical significance was considered when *p* < 0.05.

### 2.8. Cell Staining

Human gingival cells (passage 5) were seeded into a 6-well plate at the density of 40000 cells/well in complete DMEM. GD extract was dissolved in complete DMEM at the concentration of 250 *µ*g/mL. Cells were left in the CO_2_ incubator for 1 day before treatment with GD extract for 10 days. For CDr3 staining, cells were incubated with 5 *µ*M CDr3 in complete DMEM for 1 h at 37°C. Then, the cells were rinsed with incomplete DMEM for 5 minutes 3 times. Cells were covered with incomplete DMEM before image acquisition. The brightfield and fluorescence images were acquired on the OLYMOUS DP71 microscope using DP controller software. For cell morphology observation, cells were fixed in 4% formaldehyde for 10 minutes at room temperature. After that, 4% formaldehyde was discarded and replaced with methylene blue solution for 10 minutes. Methylene blue solution was washed out with tap water overnight, and cell morphology was observed under the inverted microscope.

### 2.9. Apical Papilla Cell Culture

Apical papilla cells contained higher number of mesenchymal stem cells; thus, this cell type was selected for transcriptomic analysis and to confirm the neuronal differentiation instead of human gingival cells. Molar teeth were collected in routine tooth extraction according to a protocol approved by the Ethics Committee, Faculty of Dentistry, Chiang Mai University (68/2019). Apical papilla cells were obtained from apical papilla tissues of noncarious molars. The 18–25-year-old healthy patients were recruited. The apical papilla tissues were detached and digested by using 3 mg/mL collagenase I and 4 mg/mL dispase II for 45 minutes at 37°C. Cells were cultured in complete *α*-modified Eagle medium (*α*-MEM) at 37°C and 5% CO_2_. Cells at the third passage with 80% confluent were used. Apical papilla cells were seeded into 6 well plates at the density of 50000 cells per well and maintained in 5% CO_2_ at 37°C for 24 hours to allow cell attachment. In the next day, medium was discarded and replaced with complete DMEM containing 250 *µ*g/mL GD water extract. Apical papilla cells seeded into 6-well plates at cell density of 50000 cells per well cultured in plain complete DMEM were used as control groups. Cultured medium was changed every 3 days. Cells were treated for 10 days. Total RNA extraction was performed following the instruction from NucleoSpin Kit. RNA samples were analyzed by Illumina platform, Novogene, Hong Kong. Differentially expressed genes between the control sample and test sample at 10 days culture were analyzed by log2 fold change of fragments per kilobase million (FPKM) value of treated sample compared with the FPKM value of control sample with adjusted *p* value (padj) < 0.05. Gene ontology (GO) was analyzed according to http://www.geneontology.org/. GO terms with padj <0.05 are significant enrichment.

## 3. Results

### 3.1. Extraction

Fresh GD and dried powder of GD water extract are shown in Figure 1. GD water extract of 11.92 g was obtained when 700 grams of fresh GD was used in the extraction, and % yield was equal to 1.7%. Total phenolic content of GD water extract at the concentration of 5000 *µ*g/mL was equivalent to 310.9 *µ*g/mL standard gallic acid.

### 3.2. DPPH Assay

The percentage inhibitions for DPPH assay are given in Figure 2. At all concentrations (0.031–1.0 mg/mL), GD extract at 5000 *µ*g/mL showed 39.2% DPPH radical scavenging activity.

### 3.3. The Composition of GD Extract by UHPLC-QTOF-MS/MS Analysis

Mass spectrum of the active components is shown in Figure 4S. The important chemical constituents in GD extract are shown in Table 1 including eight phenolic compounds and one flavonoid. Phenolic acids in GD extract include (1) neochlorogenic acid, (2) chlorogenic acid, (3) cryptochlorogenic acid, (4) 3-O-feruloylquinic acid, (5) 3-p-coumaroylquinic acid, (6) 4-O-feruloylquinic acid, (7) rutin, and (8) 3,4-dicaffeoylquinic acid. Flavonoid in GD extract was kaempferol-3-O-robinobioside.

### 3.4. MTT Assay

**Figure 3:**
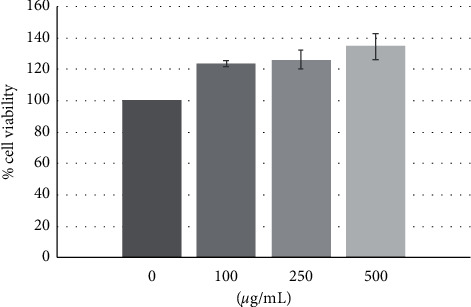
MTT assay. Treatment with 100, 250, and 500 *µ*g/mL GD water extract showed that human gingival cells viability at 48 hours was not different from control.

GD extract at the concentrations of 0, 100, 250, and 500 *µ*g/mL did not affect cell viability at the 48-hour culture period (Figure 3).

### 3.5. Cell Staining

**Figure 4:**
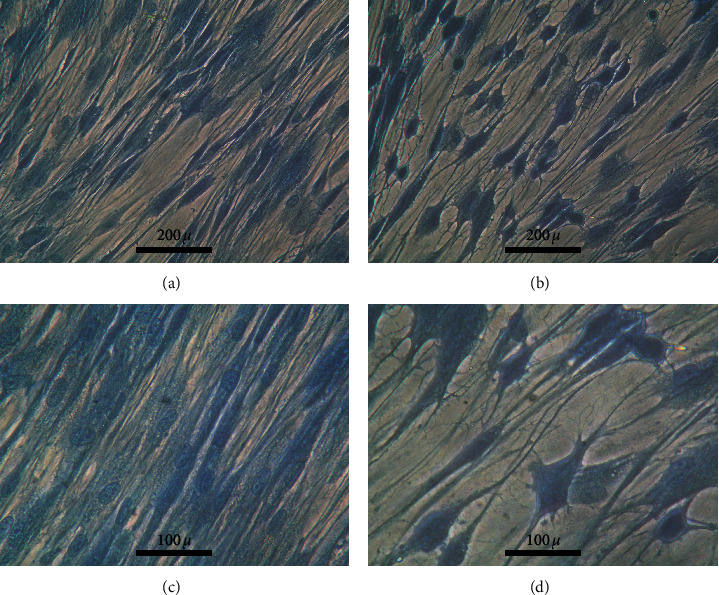
Cell morphology. (a) and (c) Human gingival cells cultured in complete DMEM for 10 days. (b) and (d) Human gingival cells cultured in complete DMEM supplemented with GD extract at the concentration of 250 *μ*g/mL for 10 days.

**Figure 5:**
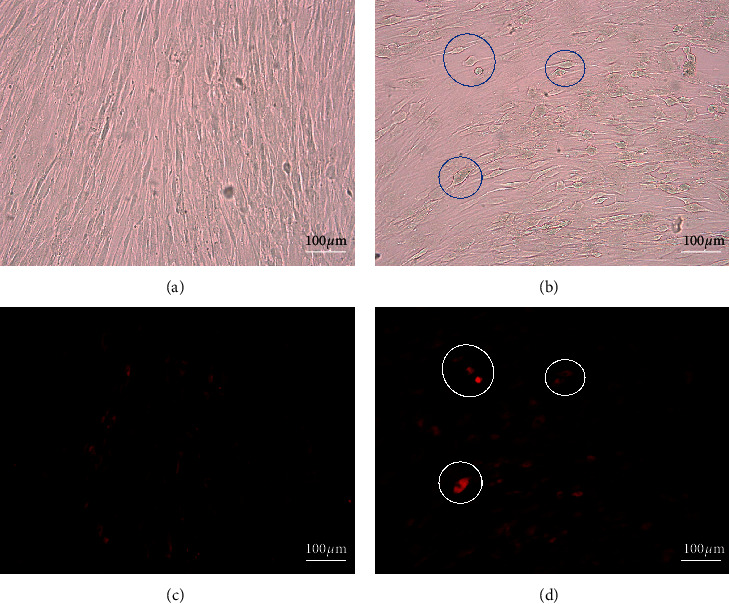
Cdr3 staining. (a) and (c) Brightfield vs. fluorescent capture of human gingival cells cultured in complete DMEM for 10 days. (b) and (d) Brightfield vs. fluorescent capture of human gingival cells cultured in complete DMEM supplemented with GD extract at the concentration of 250 *μ*g/mL for 10 days. Fluorescent signals in the control and treatment groups were detected with exposure time = 130 sec.

Cell staining with methylene blue (Figure 4) indicated that cells in the control group proliferated to form monolayer and cells treated with 250 *µ*g/mL showed less density than the control group. The morphologies of cells were different between the control and test. Cells treated with 250 *µ*g/mL GD extract (Figure 5(d)) showed the stronger fluorescent signal compared to control (Figure 5(c)). CDr3 was a membrane-permeable fluorescent probe that selectively labeled live primary and pluripotent stem cell-derived neural progenitor cells.

### 3.6. Real-Time PCR

**Figure 6:**
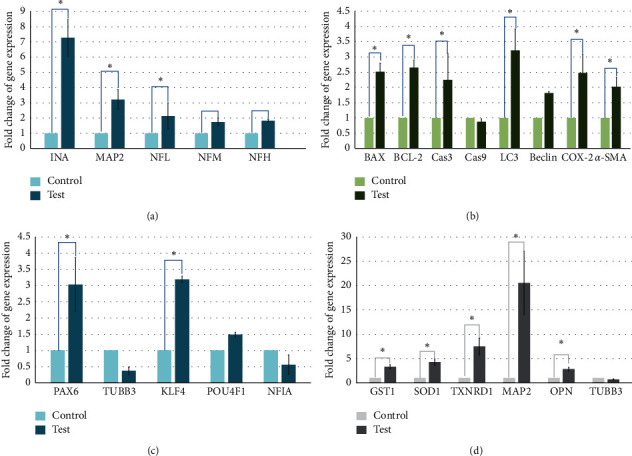
RT-PCR. (a) and (c) Human gingival cells treated with 250 *µ*g/mL of GD extract showed upregulation of *PAX6*, *KLF4*, *INA*, *MAP*2, and *NFL* genes compared to control at 10 days. (b) Human gingival cells treated with 250 *µ*g/mL of GD extract showed significant upregulation of *BAX*, *BCL-2*, *CAS3*, *LC3*, *COX-2*, and *α-SMA* genes compared to control at 3 days treatment. (d) Human gingival cells treated with 250 *µ*g/mL of GD extract showed upregulation of *PAX6*, *KLF4*, *INA*, *MAP2*, and *NFL* genes compared to control at 10 days ( ^*∗*^represented *p* ≤ 0.05).

At 3 days treatment, human gingival cells treated with 250 *µ*g/mL of GD extract showed significant upregulation of *BAX*, *BCL-2*, *CAS3*, *LC3*, *COX-2*, and *α-SMA* genes compared to control (Figure 6(b)). At 10 days, human gingival cells treated with 250 *µ*g/mL of GD extract showed upregulation of *PAX6*, *KLF4*, *INA*, *MAP2*, and *NFL* genes compared to control (Figures 6(a) and 6(c)). At 14 days, human gingival cells treated with 250 *µ*g/mL of GD extract showed upregulation of *GST1*, *SOD1*, *TXNRD1*, *MAP2*, and *OPN* genes compared to control (Figure 6(d)).

### 3.7. Transcriptomic Assay in Apical Papilla Cells

**Figure 7:**
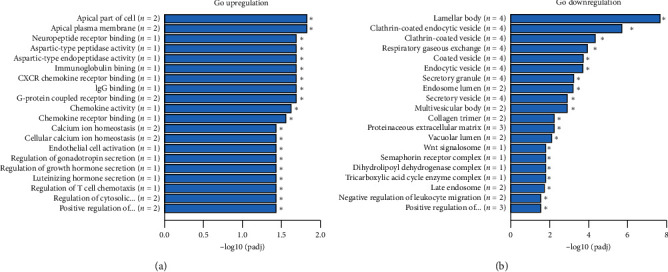
Gene ontology analysis. Graphs showed upregulation and downregulation of various sets of genes in apical papilla cells treated with 250 *µ*g/mL of GD extract for 10 days.

Apical papilla cells treated with 250 *µ*g/mL of GD extract showed a significant change of 40 genes compared to control cells. Upregulation of 13 genes and downregulation of 27 genes are presented in Table 2S. Moreover, increased expression of INA was also found in apical papilla cells treated with 250 *µ*g/mL of GD extract. Gene ontology analysis indicated downregulation of various sets of genes that involved with vesicle formation and upregulation of various receptors' binding activities (Figure 7).

### 3.8. Confirmation of INA Gene by Real-Time PCR

**Figure 8:**
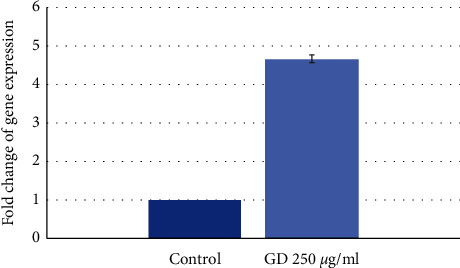
RT-PCR result in apical papilla cells treated with 250 *µ*g/mL of GD extract for 10 days. The *INA* gene expression in duplicate experiments showed that the expression of *INA* increased by 4.6 times compared with the control group at 10 days. The real-time PCR result of *INA* gene expressions was correlated with the result from RNA sequencing. Replicate control samples and test samples are presented in the form of mean ± SD.

Apical papilla cells were treated with 250 *µ*g/mL of GD water extract for 10 days. Replicate controls and tests were prepared. *INA* gene showed 4.6-fold increased expression compared to control (Figure 8). The expression of *INA* gene was correlated with the result from next-generation sequencing.

## 4. Discussion

In real life, GD was used as food, and the extraction method in this study was similar to the cooking process in kitchens of Asian people. The main active compounds of GD water extract were phenolic acids and flavonoids which possessed antioxidant capacity with no effect on cell viability at 48 hours. In China, GD was well-known as medicinal herb for diabetes mellitus patients. The MS result from our study found chlorogenic acid isoforms which possessed benefit actions in metabolism. Chlorogenic acid improved glucose and lipid metabolism by activation of AMP-activated protein kinase (AMPK) leading to suppression of hepatic glucose production and fatty acid synthesis [19]. Chlorogenic acid promoted a significant reduction of plasma glucose peak during the oral glucose tolerance test by attenuating intestinal glucose absorption which made it could be used as a glycemic index lowering agent and a compound for reducing the risk of developing type 2 diabetes [20]. Neochlorogenic acid was a phenolic compound which could be isolated from mulberry leaf, and it presented anti-inflammatory effects for the treatment of acute pneumonia [21]. Cryptochlorogenic acid was also known as 4-caffeoylquinic acid or 4-O-(e)-caffeoylquinate. This compound was previously reported in other plants such as mulberry leaves and *Chrysanthemum coronarium* [22, 23]. 3,4-Dicaffeoylquinic acid and 4,5-dicaffeoylquinic acid exhibited significant inhibitory activities against *α*-glucosidase [10]. One of flavonoid-phenolic compounds found in GD water extract was rutin because it could be considered as phenolic compound and flavonoid. Rutin was reported to possess antidiabetic effects and bone protective effects [24, 25]. Rutin has also been shown in many previous studies to exert neuroprotective effects which might be used as a natural therapy for Alzheimer's disease [26, 27]. Previous study by Banudevi et al. indicated that rutin protected differentiated neuronal cells by enhancing apoptosis through the modulation of levels of BCL-2, caspase3, surviving and by its antioxidant activity via stress-related proteins, JNK and p38 MAPK [28]. Neural degeneration was the important complication that occurred in diabetes mellitus patients. It has been reported that diabetes is also considered as a risk factor for hearing loss with possible mechanisms of vascular disease, neuropathy, and oxidative stress. Chronic inflammation could be a cause that lead to neurological damage and hearing loss [29–31]. Retinal degeneration was a major problem in people with diabetes. This may be due to changes of the capillary system in people with diabetes [32]. Changes in axons, especially distal terminals, were associated with progressive loss of synthesis and export of neurofilament polymers, which were essential structural scaffolds of the axon. Reduced mRNA expression encoding neurofilament has been proposed to underlie the loss of neurofilament polymers [33]. While other studies about GD focused on the antihyperglycemic effect, there were evidences that suggested that some herbal extracts could promote neural regeneration. For, example, the antioxidative effects of *Lycium barbarum* polysaccharides could promote nerve regeneration following cavernous nerve crush injury [34]. *Citrullus colocynthis* could reduce diabetic polyneuropathy pain in patients with painful diabetic polyneuropathy [35]. These evidences raised the hypothesis about neural regenerative effects of GD extract. Upregulation of some neuronal genes, especially, *INA* gene followed by *MAP2* and *NFL* genes indicated that GD water extract could enhance axon regeneration.

Upregulation of *BAX*, *BCL-2*, *CAS3*, and *LC3* at 3 days culture indicated the involvement of programmed cell death and autophagy which were the early processes for regulating the final number of mature neurons integrated into neural circuits [36]. Relationship between autophagy and apoptosis was linked through the action of Beclin 1 [37]. Inhibition of caspase3 could induce autophagy; meanwhile, inhibition of autophagy by caspase-3 through cleavage of Beclin 1 supported the relationship between autophagy and apoptosis. Beclin 1 binding to antiapoptotic BCL-2 negatively regulated apoptosis. BCL-2 could also function as an antiautophagic protein as its interaction with Beclin 1 inhibits autophagy [38]. Caspase-3 activation was normally considered as one of the last steps in cell death. Although caspase3 was a key protein in apoptosis execution, evidence also indicated a possible nonapoptotic role for this enzyme. Caspase3 could promote neuronal differentiation through the activation of one or more signaling pathways by cleavage of protein kinases involved in cell differentiation [39].

Increased expression of *α-SMA* might indicate epithelial–mesenchymal transition (EMT). EMT was important in direct cell fate conversion in addition to reprogramming, embryonic development, and cancer progression [40]. EMT was critical for biological processes involving cell migration, such as gastrulation, neural crest delamination, and invasion and metastasis of carcinoma cells [41, 42]. Interplay between autophagy and EMT influences cell fate [43]. *COX-2* expression could happen during acquisition of an epithelial–mesenchymal transition (EMT) phenotype [44]. *COX-2* was transcriptionally upregulated and caused increased intracellular prostaglandin *E*_2_ levels, which promoted migration [45].

At 10 days, the expression of *PAX6* and *KLF4* was dominant. The transcription factor *Pax6* was essential for neural stem cell proliferation, multipotency, and neurogenesis. Increasing *Pax6* levels drives the system towards neurogenesis with decreasing of self-renewal and turning on a genetic program for making neurons [46]. Klf4 was expressed strongly in early granule cell progenitor development but tailsoff considerably by the end of embryonic development. *KLF4* was also coexpressed with *Pax6* in neural stem cells [47]. Krüppel-like factor 4 (KLF4) was expressed in neural stem cells and controls axonal regeneration [48]; unsurprisingly, upregulation of *INA*, *MAP2*, and *NFL* was observed. *INA* was encoded for alpha-internexin which was a 66 kDa neuronal intermediate filament protein found most abundantly in the neurons of the nervous systems during early development [49]. Alpha-internexin was a fourth subunit of neurofilaments in the adult CNS [50] that played an important role in neurite outgrowth and regulates the expression of neurofilaments during neuronal development [51]. In the present study, increased expression of neurofilaments was observed (neurofilament light, neurofilament medium, and neurofilament heavy). However, only neurofilament light was considered upregulated because its expression was 2-fold higher than the control. This evidence was correlated to the previous literature from Braissant who mentioned that NFL appeared first at the start of neuronal differentiation, overlapping with *α*-internexin. NFM followed NFL shortly after when neurite elongation starts [52]. Alpha-internexin might involve with the construction of the postsynaptic density backbone and provided linker sites for various postsynaptic density scaffold protein complexes [53]. MAP2 is a neuron-specific protein that stabilizes microtubules in the dendrites of postmitotic neurons [54]. Neurofilaments (NFs) were intermediate filaments with a diameter of 10 nm and transcription occurred during axonal regeneration [55]. They were abundant in axons and essential for the radial growth of axons during development [56]. MTT assay demonstrated that the cell viability was slightly increased when treated with GD extract for 48 hours; however, at 10 days culture, the treated cells showed less density than the control group (Figure 4) because cells switched from proliferation to the differentiation stage. The expression of apoptotic and autophagic genes together with neuronal specific genes might inhibit proliferation and promote differentiation.

Pou4f1 (or *BRN3A*) was slightly upregulated. Pou4f1, a member of the POU (Pit-Oct-Unc) family of transcription factors, was shown to be more strongly expressed in a single class of type I spiral ganglion neurons regulating sensory afferent projections to the central targets [57, 58]. *NIFA* was downregulated in this study which indicated negative astrocytic differentiation because *NFIA* promotes migration and differentiation of astrocyte precursors [59]. This study could not detect *Olig2* and *GFAP* in any timepoint; thus, no astrocyte or oligodendrocyte differentiation was observed.

At 14 days, the expression of *MAP2* was increased to 20 folds. However, *TUBB3* gene did not show upregulation. The result showed upregulation of osteopontin (*OPN*). Previous study indicated positive effects of OPN on survival, proliferation, migration, and neuronal differentiation of *NSC* [60]. Moreover, the present study presented the protective effect of GD extract because upregulation of antioxidant enzymes *SOD1*, *GST1*, and *Txnrd1* was detected at 14 days culture. A major role for *Txnrd1* in postnatal proliferation of granule cells was detected in development of the cerebellum. *Txnrd1* might play a major role of the cell proliferation in neuronal precursor cells [61].

The results of gene expression indicated neurogenesis. However, the expressions of *MAP2*, *TUBB3, NFM*, and Pou4f1 were not upregulated but the expression of *INA* was observed at 5 days culture (Figure 5S; fold change = 2.5). The staining with Cdr3 was confirmed at 10 days culture. This result supported that some populations of cells were in the state of neuronal progenitors. Cdr3 bound to fatty acid binding protein 7 (FABP7), which is highly expressed in neural stem cells and localized in the cytoplasm [62, 63].

*TUBB3* was not upregulated at any timepoint. *TUBB3* gene was also expressed in human gingival cells because *TUBB3* gene was normally expressed at high levels in cells of neuronal origin [64]. Human gingiva contained neural crest-derived stem cells which also expressed *TUBB3* gene [65]. Another source of neural crest-derived stem cells was apical papilla tissue [66]. Screening with RNA sequencing supported the results from human gingival cells because upregulation of *INA* gene was found. Other upregulated genes that might relate to neuronal differentiation were *MPP4* and *KISS1*. Membrane protein, palmitoylated-4 (*MPP4*) was important as a regulator of synaptic plasticity, leading to changes in synaptic strength [67]. *KISS1* gene encoded kisspeptin neuropeptides which were potent stimulators of GnRH secretion [68]. The GO term neuropeptide receptor binding supported that neuronal differentiation might occur which needed more detailed studies. The further study employing other techniques was continued in our laboratory which should be reported later on.

## 5. Conclusions

The study highlighted the possibility of GD water extract on neuronal protection and neuronal differentiation. In diabetic patients, neurodegenerations were important complications. GD might have neural regeneration effects while it was used as functional food to improve hyperglycemia in diabetes mellitus which increased the benefit to patients. GD increased neuronal specific gene expression in human gingival cells, i.e., *PAX6*, *INA*, *MAP2*, and *NFL* together with neuronal morphology and slightly positive Cdr3. GD contained the important compounds such as rutin and chlorogenic acid isoforms which possessed neuroprotective and antidiabetic effects. Additionally, GD contained antioxidant activity which could help to eliminate oxidative stress that normally happen in metabolic diseases. This study provided the basis for further investigations into the function of GD water extract in neuronal differentiation of human primary cells. The study about transcriptomic and proteomic change in human gingival cells treated with GD water extract is continuing in our laboratory and will be reported in the future.

## Figures and Tables

**Table 1:** Active compounds found in GD water extract.

Peak	Class	Chemical formula	Name of compound	Molecular weight	Cas
1	Phenolic acid	C_16_H_18_O_9_	Neochlorogenic acid	354.31 g/mol	906-33-2
2	Phenolic acid	C_16_H_18_O_9_	Chlorogenic acid	354.31 g/mol	327-97-9
3	Phenolic acid	C_16_H_18_O_9_	Cryptochlorogenic acid	354.31 g/mol	905-99-7
4	Phenolic acid	C_17_H_20_O_9_	3-O-Feruloylquinic acid	368.3 g/mol	1899-29-2
4	Phenolic acid	C_16_H_18_O_8_	3-p-Coumaroylquinic acid	338.31 g/mol	87099-71-6
5	Phenolic acid	C_17_H_20_O_9_	4-O-Feruloylquinic acid	368.3 g/mol	905-99-7
6	Phenolic acid	C_27_H_30_O_16_	Rutin	610.5 g/mol	153-18-4
7	Phenolic acid	C_25_H_24_O_12_	3,4-Dicaffeoylquinic acid	516.4 g/mol	14534-61-3
7	Flavonoids	C_27_H_30_O_15_	Kaempferol-3-O-robinobioside	594.5 g/mol	17297-56-2

## Data Availability

The data used to support the findings of this study are available from the corresponding author upon request.

## Abbreviations

DPPH: 2,2-Diphenyl-1-picrylhydrazyl
MTT: 3-(4,5-Dimethylthiazol-2-yl)-2,5-diphenyltetrazolium bromide
MS: Mass spectrometry
QTOF: Quadrupole time-of-flight
UHPLC: Ultrahigh performance liquid chromatography
*BAX*: BCL- (B cell lymphoma-) associated X
*BCL-2*: B cell lymphoma 2
*CAS3*: Caspase-3
*COX-2*: Cyclooxygenase-2
*α-SMA*: Alpha-smooth muscle actin
*PAX6*: Paired box gene 6
*KLF4*: Krüppel-like factor 4
*INA*: Alpha-internexin
*MAP2*: Microtubule-associated protein 2
*NFL*: Neurofilament light polypeptide
*NFM*: Neurofilament medium polypeptide
*NFH*: Neurofilament heavy polypeptide
*GST1*: Glutathione-S-transferase I
*SOD1*: Cu/Zn superoxide dismutase
*TXNRD1*: Thioredoxin reductase 1
*TUBB3*: Tubulin beta-3 class III
IRS1: Insulin receptor substrate 1
GLUT1: Glucose transporter 1.

## Supplementary Materials

Supplementary Materials The additional information about the location of the obtained material and plant material processing are available in Supplementary file (Figures 1S and 2S). The Supplementary file also provided the additional results about antioxidant assay of GD water extract (Figure 3S), mass spectrum of each active compound (Figure 4S), gene expression in human gingival cells (Figure 5S), and differential gene expression in apical papilla cells (Table 2S). The primer sequences used in this study are available in Supplementary file (Table 1S).
