# Supplementary material for: Gynura divaricata Water Extract Presented the Possibility to Enhance Neuronal Regeneration
Source: Evid Based Complement Alternat Med. 2021 Feb 17;2021:8818618. doi: 10.1155/2021/8818618 (PMC7904343; doi:10.1155/2021/8818618)
Supplement: Supplementary Materials — The additional information about the location of the obtained material and plant material processing are available in Supplementary file (Figures 1S and 2S). The Supplementary file also provided the additional results about antioxidant assay of GD water extract (Figure 3S), mass spectrum of each active compound (Figure 4S), gene expression in human gingival cells (Figure 5S), and differential gene expression in apical papilla cells (Table 2S). The primer sequences used in this study are available in Supplementary file (Table 1S). [file 8818618.f1.docx]

**Supplementary data**

**
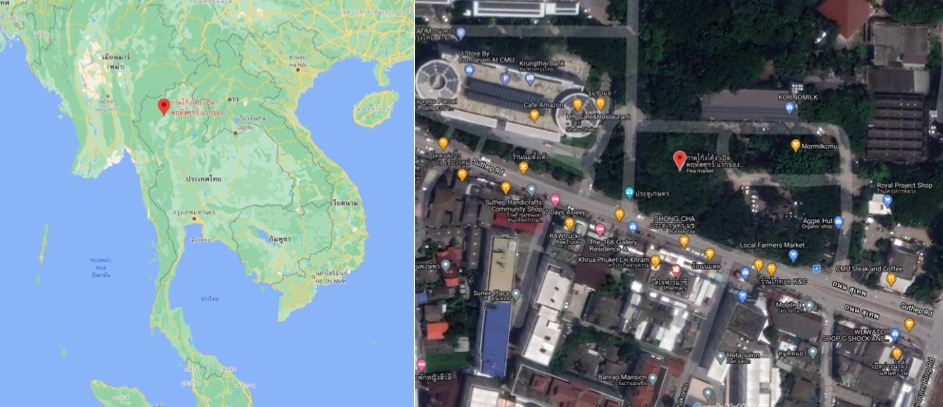
**

**FIGURE 1S.** GPS location of the obtained plant material.

**
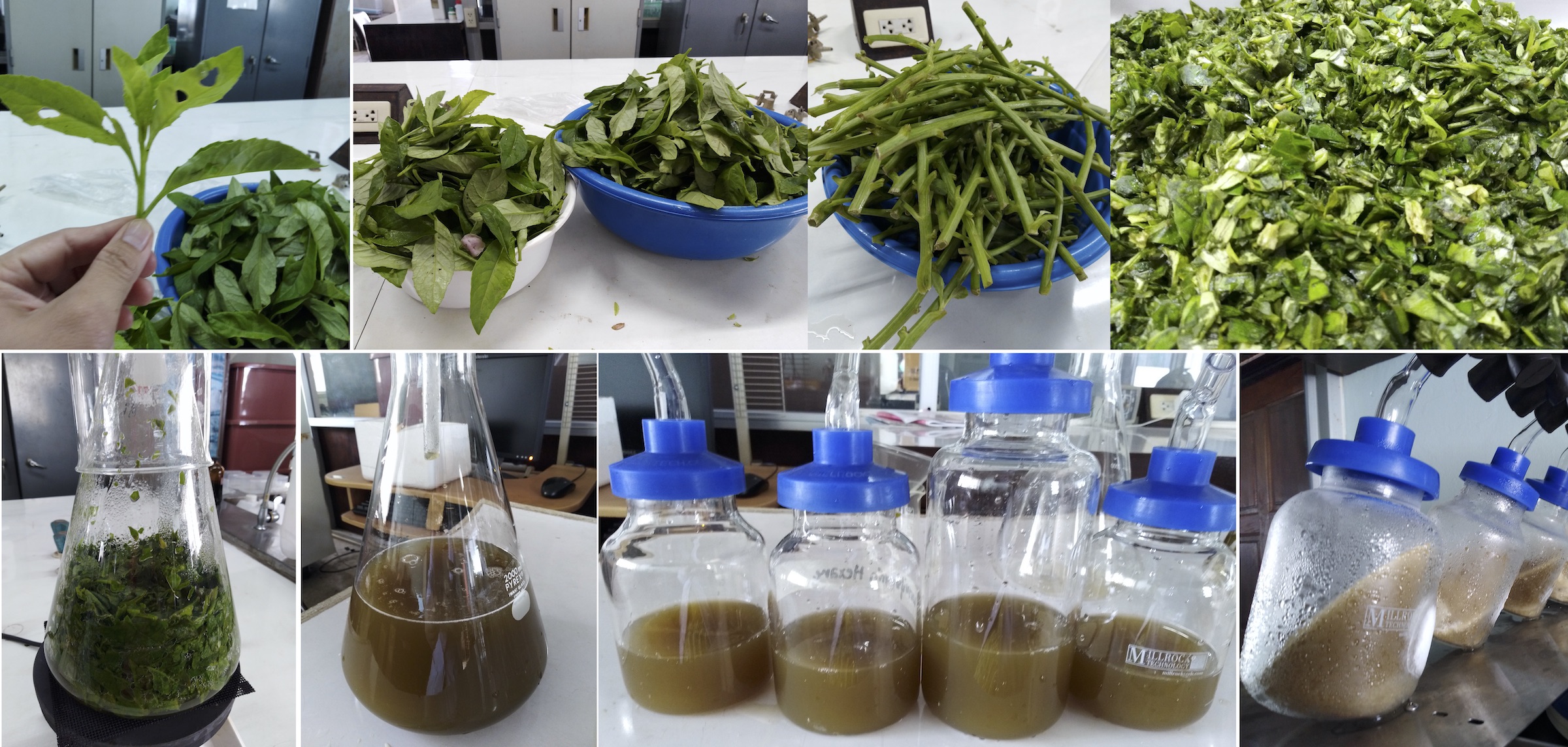
**

**FIGURE 2S. Plant material processing.** The leaves of *GD* were used in this study and the stems were discarded. After purchasing, *GD* leaves were cleaned in tab water and 700 grams of *GD* leaves were cut into small pieces and boiled in distilled water 1,400 mL for 5 minutes. After that, the boiled solution was cooled down at 4 °C overnight. The next day, the solution was filtered through white gauze. The filtrate was aliquoted in lyophilized flasks and frozen at -20 °C before lyophilized into powder for 7 days.

**Antioxidant capacity (ABTS assay)**

Dried GD water extract was dissolved in distilled water to the concentrations of 1000, 500, 250, 125, 62.5 and 31.25 µg / mL. 30 µL of GD solution was mixed with working ABTS reagent 2970 µL and left for 1 min. OD was measured at 734 nm using distilled water to set 0. Standard Trolox at the same concentration was used as positive control. Working ABTS reagent was prepared from: 14 mmol/L ABTS reagent mixed with 5 mmol/L potassium persulfate solution (1:1). Working ABTS reagent should have absorbance around 0.700 ± 0.02 (0.68–0.72) at 734 nm. Triplicate assays were performed for each dilution. The %inhibition of each dilution **was presented in the form of mean ± SD.**

$$\%inhibition=\frac{Absorbance control- Absorbance test}{Absorbance control} 100$$

**
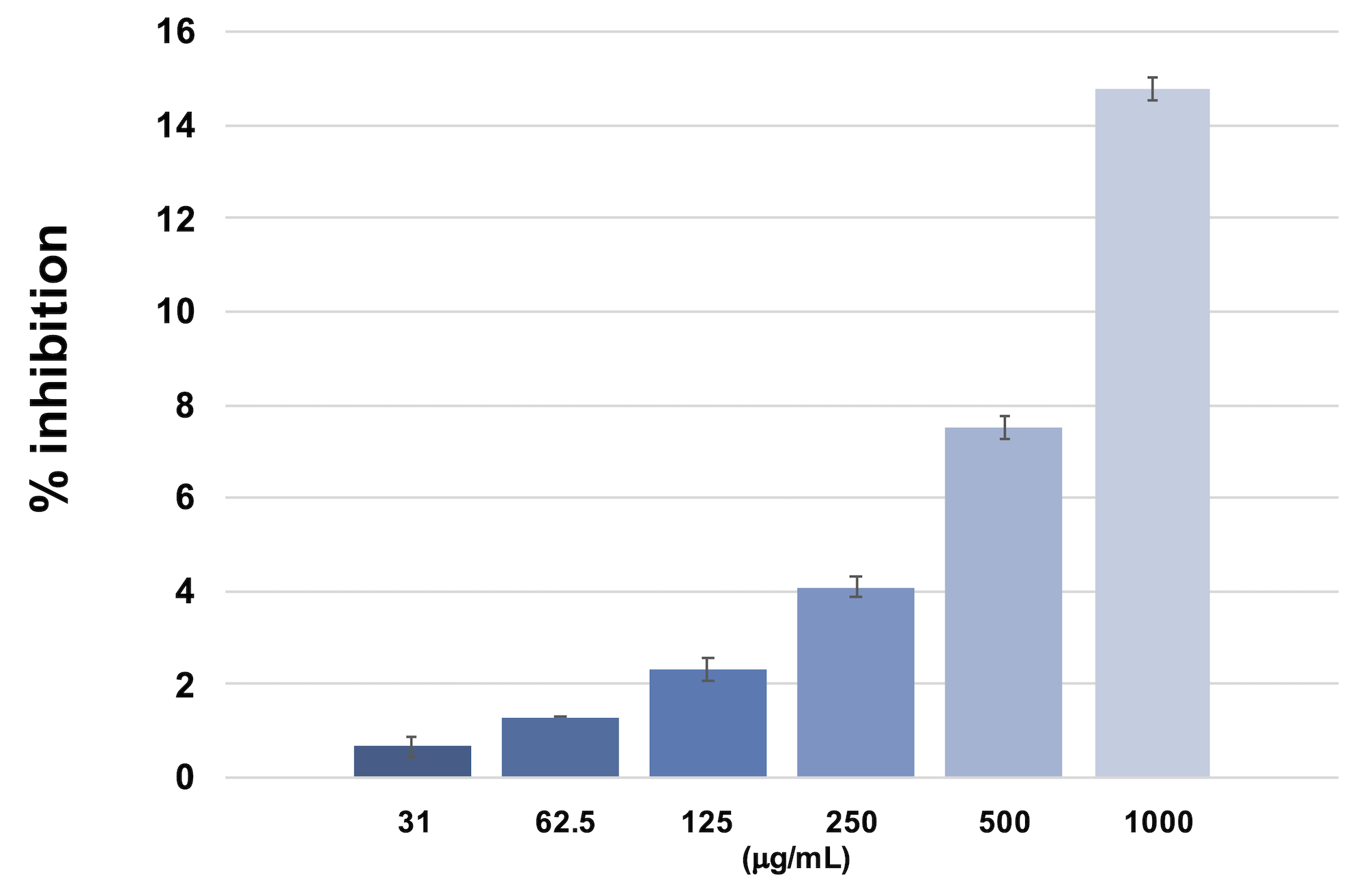
**

**FIGURE 3S. ABTS assay.** Antioxidant activity of *G. divaricata* extract at various concentrations. *GD* extract at 1000 μg/mL showed 15% ABTS radical scavenging activity.

**
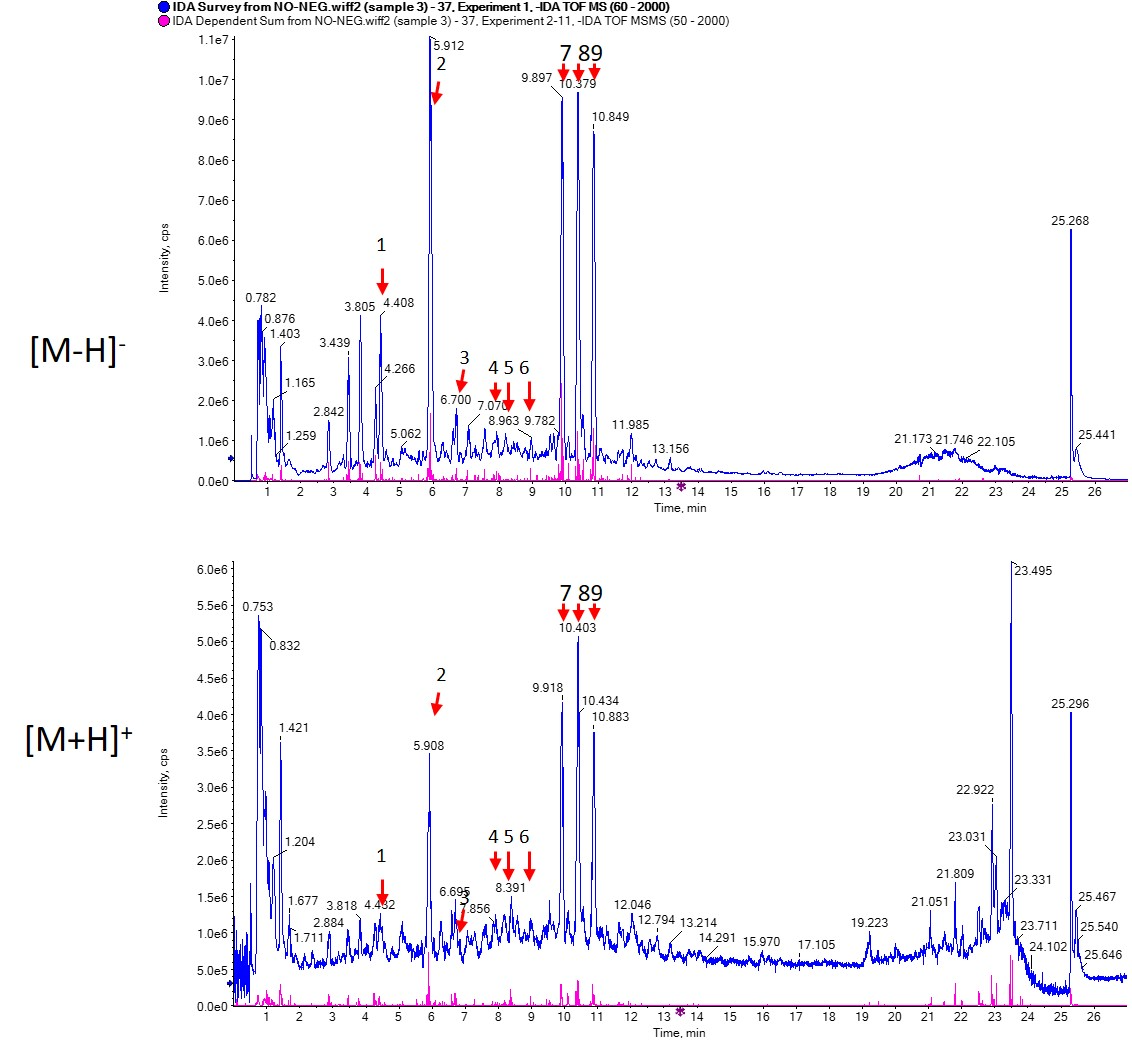
**

**FIGURE 4S. The mass spectra of *G. divaricata* extract.** m/z of negative ion mode [M-H]^-^ and positive mode [M+H]^+^ ions generated by electrospray were detected by positive and negative full scans.

**
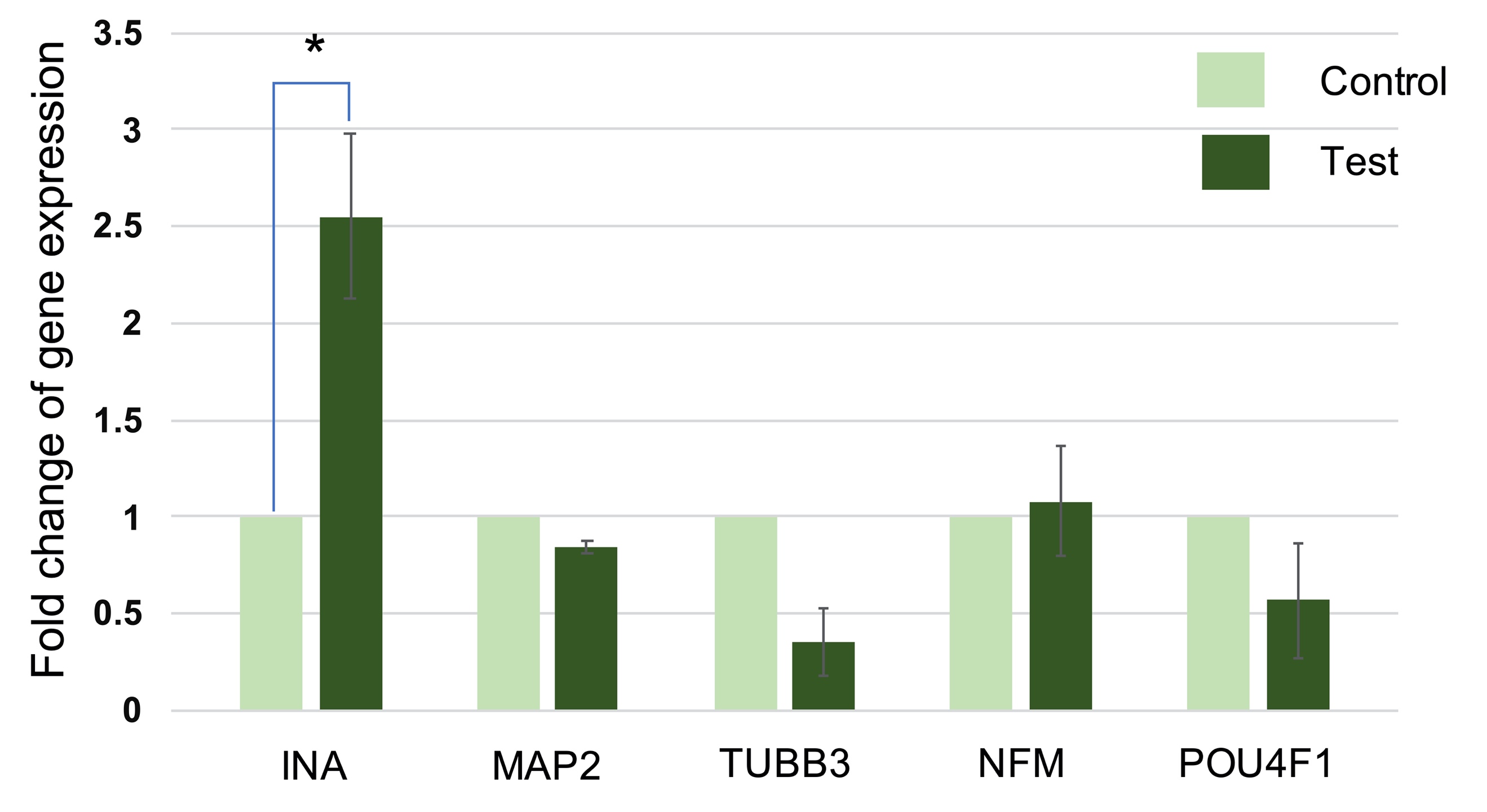
**

**FIGURE 5S: RT-PCR.** Human gingival cells treated with 250 µg/mL of *GD* extract showed only up-regulation of *INA* gene compared to control at 5 days (* = p < 0.05).

**Table 1S Primer sequences**

| ***Genes*** | **Sequence (5'->3')** |
| --- | --- |
| *1. GAPDH* | (F)AAATCCCATCACCATCTTCCAGGAGC |
|  | (R) CATGGTTCACACCCATGACGAACA |
| *2. INA* | (F) GCTACCAGGACAGCATTGG |
|  | (R) ATTCAGCCCCGAGATGCTTA |
| *3. POU4F1* | (F) CACCCTCCCTGAGCACAAGT |
|  | (R) AGGCTAGGGGACAGCAAAGG |
| *4. NFH* | (F) GCCAAGGTGGAGGTGAAGGA |
|  | (R) TGGTCTGTGCTGGAGGATTTTT |
| *5. NFM* | (F) TGCTCCCTCCTCAGTCTTTGG |
|  | (R) TCGTTTATTGTTTTTGGCTCAGTTG |
| *6. NFL* | (F) TGGCCTTGGACATCGAGATTGCA |
|  | (R) GCTTCTCCTTCAGAGGGGGGC |
| *7. BAX* | (F) GGTTGTCGCCCTTTTCTA |
|  | (R) CGGAGGAAGTCCAATGTC |
| *8. BCL-2* | (F) GATGTGATGCCTCTGCGAAG |
|  | (R) CATGCTGATGTCTCTGGAATCT |
| *9. caspase-9* | (F) ATGGACGAAGCGGATCGGCGGCTCC |
|  | (R) GCACCACTGGGGGTAAGGTTTTCTAG |
| *10. caspase-3* | (F) TTCAGAGGGGATCGTTGTAGAAGTC |
|  | (R) CAAGCTTGTCGGCATACTGTTTCAG |
| *11. Beclin1* | (F) GGCTGAGAGACTGGATCAGG |
|  | (R) CTGCGTCTGGGCATAACG |
| *12. LC3-II* | (F) GAGAAGCAGCTTCCTGTTCTGG |
|  | (R) GTGTCCGTTCACCAACAGGAAG |
| *13. PAX6* | (F) AGTGAATGGGCGGAGTTAT |
|  | (R) ACTTGGACGGGAACTGACA |
| *14. GSTT1* | (F) GCCACACTCTCCGTCAA |
|  | (R) TGCCAAGAAGAACGACATTCC |
| *15. TXNRD1* | (F) GAAGATCTTCCCAAGTCCTATGAC |
|  | (R) ATTTGTTGCCTTAATCCTGTGAGG |
| *16. SOD1* | (F) GTAATGGACCAGTGAAGGTGTG |
|  | (R) CAATTACACCACAAGCCAAACG |
| *17. MAP2* | (F) CCAAGGAGTCTGATTGCAGGA |
|  | (R) CCTCAACCACAGCTCAAATGC |
| *18. TUBB3* | (F) CTCAGGGGCCTTTGGACATC |
|  | (R) CAGGCAGTCGCAGTTTTCAC |
| *19. COX-2* | (F) CCCTTGGGTGTCAAAGGTAA |
|  | (R) GCCCTCGCTTATGATCTGTC |
| *20. α-SMA* | (F) TGACAATGGCTCTGGGCTCTGTAA |
|  | (R) TTCGTCACCCACGTAGCTGTCTTT |
| *21. OPN* | (F) GGACAGCCAGGACTCCATTG |
|  | (R) TGTGGGGACAACTGGAGTGAA |
| *22. NFIA* | (F) CAAGCCTCCAACCACATCAAC |
|  | (R) CTGTTTGACCACGATGTTTGCT |
| *23. Klf4* | (F) GAAATTCGCCCGCTCCGATGA |
|  | (R) CTGTGTGTTTGCGGTAGTGCC |

**Table 2S Differential gene expression**

| **GeneName** | **GeneDescription** | **log2FoldChange** | **padj** |
| --- | --- | --- | --- |
| 1. SFTPA2 | Surfactant protein A2 | -9.2758 | 3.01E-08 |
| 2. IGHG1 | Immunoglobulin heavy constant gamma 1 | -8.5622 | 1.62E-05 |
| 3. SFTPB | Surfactant protein B | -8.0837 | 0.000511 |
| 4. SEMA6B | Sema domain transmembrane domain (TM) and cytoplasmic domain (semaphorin) 6B | -7.3628 | 0.026196 |
| 5. CHRNA5 | Cholinergic receptor nicotinic alpha 5 (neuronal) | -7.2324 | 0.042602 |
| 6. SFTPC | Surfactant protein C | -6.1909 | 1.08E-07 |
| 7. SFTPA1 | Surfactant protein A1 | -5.536 | 3.88E-05 |
| 8. SCARA5 | Scavenger receptor class A member 5 (putative) | -4.2176 | 2.24E-06 |
| 9. WNT2 | wingless-type MMTV integration site family member 2 | -3.3834 | 0.000101 |
| 10. RASD2 | RASD family member 2 | -3.2704 | 0.001688 |
| 11. C5AR2 | Complement component 5a receptor 2 | -3.2588 | 0.002028 |
| 12. OGDHL | Oxoglutarate dehydrogenase-like | -3.1288 | 0.002028 |
| 13. DMKN | dermokine | -3.001 | 1.62E-05 |
| 14. GLDN | gliomedin | -2.9616 | 0.034908 |
| 15. NTRK1 | Neurotrophic tyrosine kinase receptor type 1 | -2.8124 | 0.035831 |
| 16. CPXM1 | Carboxypeptidase X (M14 family) member_1 | -2.7214 | 0.008821 |
| 17. MGP | Matrix Gla protein | -2.6451 | 0.046151 |
| 18. CXCL12 | Chemokine (C-X-C motif) ligand 12 | -2.5749 | 2.79E-05 |
| 19. EFNB3 | Ephrin B3 | -2.5513 | 0.034908 |
| 20. ADAM11 | ADAM metallopeptidase domain 11 | -2.4638 | 0.001543 |
| 21. DDIT4L | DNA-damage-inducible transcript 4-like | -2.306 | 0.000605 |
| 22. SORBS2 | Sorbin and SH3 domain containing_2 | -2.2385 | 0.009193 |
| 23. NR4A3 | Nuclear receptor subfamily 4 group A member 3 | -2.2107 | 0.020897 |
| 24. PLXNC1 | Plexin C1 | -2.2017 | 0.002472 |
| 25. APOE | Apolipoprotein E | -2.1686 | 0.011225 |
| 26. PCDH10 | Protocadherin 10 | -2.0935 | 0.034908 |
| 27. FOS | FBJ murine osteosarcoma viral oncogene homolog | -2.0135 | 0.041276 |
| 28. TMEM171 | Transmembrane protein 171 | 1.9927 | 0.020897 |
| 29. HIST1H2BC | Histone cluster 1 H2bc | 2.0133 | 0.030665 |
| 30. SERPINB7 | Serpin peptidase inhibitor clade B (ovalbumin) member 7 | 2.0657 | 0.013632 |
| 31. CTNND2 | Catenin (cadherin-associated protein) delta 2 | 2.1352 | 0.013632 |
| 32. PIP | prolactin-induced protein | 2.1837 | 0.004918 |
| 33. INA | Internexin neuronal intermediate filament protein alpha | 2.2923 | 0.000933 |
| 34. HIST1H4H | Histone cluster 1 H4h | 2.3039 | 0.007774 |
| 35. MPP4 | Membrane protein palmitoylated 4 (MAGUK p55 subfamily member 4) | 2.4626 | 0.013632 |
| 36. ALDH3A1 | Aldehyde dehydrogenase 3 family member A1 | 2.531 | 0.028497 |
| 37. CD36 | CD36 molecule (thrombospondin receptor) | 2.5775 | 0.034908 |
| 38. KISS1 | KiSS-1 metastasis-suppressor | 2.9202 | 9.14E-05 |
| 39. HIST1H2BG | Histone cluster 1 H2bg | 3.6467 | 0.000992 |
| 40. CXCL10 | Chemokine (C-X-C motif) ligand 10 | 5.4977 | 2.89E-05 |
